# Introduced Pathogens and Native Freshwater Biodiversity: A Case Study of *Sphaerothecum destruens*

**DOI:** 10.1371/journal.pone.0036998

**Published:** 2012-05-15

**Authors:** Demetra Andreou, Kristen D. Arkush, Jean-François Guégan, Rodolphe E. Gozlan

**Affiliations:** 1 Centre for Conservation Ecology and Environmental Change, School of Applied Sciences, Bournemouth University, Fern Barrow, Poole, Dorset, United Kingdom; 2 Cardiff School of Biosciences, Biomedical Building, Museum Avenue, Cardiff, United Kingdom; 3 Argonne Way, Forestville, California, United States of America; 4 Maladies Infectieuses et Vecteurs : Écologie, Génétique, Évolution et Contrôle, Institut de Recherche pour le Développement, Centre National de la Recherche Scientifique, Universities of Montpellier 1 and 2, Montpellier, France; 5 French School of Public Health, Interdisciplinary Centre on Climate Change, Biodiversity and Infectious Diseases, Montpellier, France; Institute of Marine Research, Norway

## Abstract

A recent threat to European fish diversity was attributed to the association between an intracellular parasite, *Sphaerothecum destruens*, and a healthy freshwater fish carrier, the invasive *Pseudorasbora parva* originating from China. The pathogen was found to be responsible for the decline and local extinction of the European endangered cyprinid *Leucaspius delineatus* and high mortalities in stocks of Chinook and Atlantic salmon in the USA. Here, we show that the emerging *S. destruens* is also a threat to a wider range of freshwater fish than originally suspected such as bream, common carp, and roach. This is a true generalist as an analysis of susceptible hosts shows that *S. destruens* is not limited to a phylogenetically narrow host spectrum. This disease agent is a threat to fish biodiversity as it can amplify within multiple hosts and cause high mortalities.

## Introduction

Introduction of non-native species is known to pose high risks to native biodiversity in particular through introducing exotic virulent pathogens to naïve wild populations [1]–[4]. In freshwater ecosystems, non-native species introductions have been shown to be closely associated with human activity and the aquaculture industry [5]. Aquaculture facilities are often connected to rivers, thereby potentially increasing the risk of disease transmission from farmed fish to sympatric wildlife.

Parasite life history traits such as host specificity can heavily influence the probability of parasite transfer with invasive species [4] as well as the probability of host switch to a new naïve host. For example, generalist parasites as opposed to highly host-specific parasites are highly likely to switch hosts as they are equipped to parasitize a wide range of hosts. A wide host range ensures that the parasite can persist within a community. [6]–[7].

The decline and local extinctions of the previously widespread sunbleak *Leucaspius delineatus* in mainland Europe could represent a compelling example of the impact of both non-native species introductions and their microbial agents [6]. *Leucaspius delineatu*s is the only representative of this genus and is now on the red list of species for a range of European countries and extinct in Slovenia [8]. Gozlan et al. [6] have shown that the population decline of this native mainland European cyprinid could be linked to the introduction of the topmouth gudgeon *Pseudorasbora parva*, a non-native cyprinid originating from Asia that was accidentally introduced into Romanian aquaculture facilities [9]. In both semi-natural (pond) and laboratory experiments, Gozlan et al. [6] demonstrated that *L. delineatus* cohabited with *P. parva* failed to reproduce and that their population experienced a dramatic decline. This work has also shown *P. parva* to harbour *Sphaerothecum destruens* [6] a protistan pathogen responsible for disease outbreaks in salmonids in North America [10]–[11].

*Sphaerothecum destruens* is a member of a new monophyletic clade at the boundary of animal-fungal divergence [12] which includes other significant pathogens of amphibians, e.g., *Amphibiocystidium ranae* [13], and of birds and mammals including humans, e.g., *Rhinosporidium seeberi* [14]. Previous work has established that *S. destruens* is not host specific and that a range of salmonid species are susceptible to the pathogen [6], [15]–[16]. *S. destruens* causes chronic but steady mortality in both subadult and adult Atlantic *Salmo salar*, Chinook salmon *Oncorhynchus tshawytscha* and *L. delineatus* [6], [10]–[11], [15]–[17].

Detecting disease related mortality in the wild is biased towards pathogens causing simultaneous, short-lived high mortalities, such as the mortality patterns caused by viral infections. Chronic, steady mortality is often undetected and underreported although it can lead to equally high mortalities and devastating effects on populations. Despite the slow-growing nature of *S. destruens* in the fish after infection, parasitism ultimately results in host cell death and often causes widespread destruction of various tissues [15]–[17].

*Sphaerothecum destruens* has an extracellular, motile zoospore stage [18]–[19] which is triggered when spores are in contact with fresh water and may facilitate spread to new hosts which have been shown to be more susceptible during their reproductive period [20]. However, due to the nature of the disease (i.e. slow growing), there have been limited attempts to assess the parasite's prevalence in wild populations other than through cohabitation of wild individuals with susceptible species. Nonetheless, the presence of *S. destruens* was demonstrated in up to 32% of hatchery-produced adult late Fall run Chinook salmon returning to the Upper Sacramento River of California, USA [15] and 5% in a wild *L. delineatus* population in the UK [17].

The main concern that has arisen from the Gozlan et al. paper [6] is the risk *S. destruens* poses to European freshwater biodiversity. Its association with invasive fish species such as *P. parva*, a healthy carrier [6], [21], presents a risk of disease transfer from wild invasive populations to sympatric populations of susceptible native fish and as such could have major implications for fish conservation and aquaculture in Europe. Our objective was to determine the susceptibility of native cyprinid species (carp *Cyprinus carpio*, bream *Abramis brama* and roach *Rutilus rutilus*) to allopatric *S. destruens* and evaluate the risk posed to European fish biodiversity. In order to better elucidate the risks associated with *S. destruens*, a meta analysis of genetic distance between susceptible fish host species and susceptibility to the parasite was performed and used to assess the generalist nature of the pathogen.

## Results

**Figure 1:**
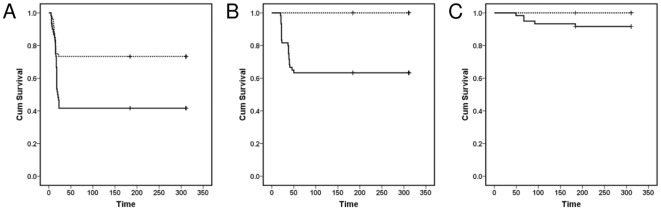
Kaplan-Meier survival curves for *Abramis brama*, *Rutilus rutilus* and *Cyprinus carpio* following infection with *Sphaerothecum destruens*. Cumulative proportion of (A) Bream *Abramis brama*, (B) Roach *Rutilus rutilus* and (C) Carp *Cyprinus carpio* surviving following exposure to *S. destruens*. Treatment fish (solid line) were exposed to an average concentration of 8.6×10^4^ *S. destruens* spores ml^−1^ whilst control fish (dotted line) were sham exposed. Time: days post exposure.

**Figure 2:**
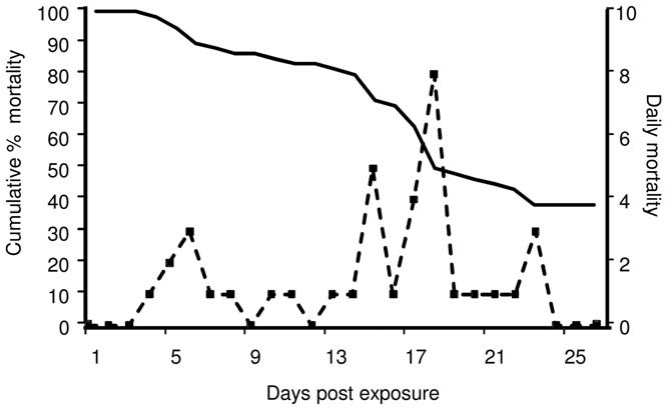
Mortality pattern in *Abramis brama* as a result of infection with *Sphaerothecum destruens*. The cumulative percentage mortality in the treatment groups (n = 60 individuals in total) and daily mortalities are presented for 26 days post exposure with *S. destruens*.

Experimental exposure to *S. destruens* led to significantly higher mortalities in *A. brama*, *C. carpio* and *R. rutilus* groups as compared to controls (Log rank test; *A. brama*: Chi-square = 10.6, d.f. = 1, *P*<0.05; *C. carpio*: Chi-square = 5.18; d.f. = 1; *P*<0.05; *R. rutilus*: Chi-square = 26.96; d.f. = 1; *P*<0.05). *A. brama* experienced high mortalities over a period of 23 days following exposure to *S. destruens* (mean mortality 53%; Figures 1, 2). The parasite was detected (by nested polymerase chain reaction [PCR]) in the kidney, liver and intestine of *A. brama* mortalities in the treatment groups with an overall prevalence of 75% (Table 1). All *A. brama* mortalities in the control group were also tested for the presence of *S. destruens* (nested PCR; kidney, liver, intestine) and were found negative for the parasite.

**Table 1:** *Sphaerothecum destruens* prevalence mortalities of *Abramis brama*, *Cyprinus carpio* and *Rutilus rutilus* exposed to the parasite via bath immersion.

Species	Organs					Overall prevalence
	K	L	I	Gi	Go	
*A. brama* (n = 32)	75 (24/32)	63 (20/32)	34 (11/32)	n/t	n/t	75 (24/32)
*C. carpio* (n = 5)	20 (1/5)	0 (0/5)	20 (1/5)	n/t	n/t	20 (1/5)
*R. rutilus* (n = 22)	5 (1/22)	5 (1/22)	5 (1/22)	0 (0/13)	0 (0/13)	5 (1/22)

Overall prevalence (%) and organ specific prevalence is provided per species. The proportion of fish testing positive for *S. destruens* is also provided. Organs tested included the kidney (K), liver (L), intestine (I), gill (Gi) and gonad (Go). n: number of mortalities. n/t: not tested for *S. destruens*.

Experimentally-exposed *C. carpio* experienced an 8% mortality rate between 49 and 92 days post exposure (d.p.e.) (Figure 1). *Sphaerothecum destruens* DNA was detected in the kidney and intestine of *C. carpio* mortalities and sampled fish of the treatment group. Parasite DNA was detected in the intestine of two out of ten *C. carpio* sampled at 28 d.p.e. resulting in 20% prevalence in these individuals and in one out of five mortalities (Table 1). Mortality in *R. rutilus* challenged with *S. destruens* was 37% (Figure 1) and the majority of mortalities occurred between 20 and 50 d.p.e. *S. destruens* DNA was detected in the kidney, liver and intestine of one of twenty-two *R. rutilus* mortalities at 23 d.p.e., resulting in a parasite prevalence of 5% in that species. Parasite DNA was not detected in the gills and gonads of the 13 *R. rutilus* mortalities analyzed (Table 1).

*Sphaerothecum destruens* DNA was not detected in the kidney, liver and intestine (by nested PCR) at six months post exposure or at the end of the experiment in both the treatment and control groups of all three cyprinids. Mean length and weight for the three species at the onset of the experiment were: 7.1 cm and 8.3 g for *A. brama*; 8.2 cm and 8.4 g for *R. rutilus*; and 7.4 cm and 7.0 g for *C. carpio*. There was no significant difference in body condition (Mann Whitney U test; *A. brama P* = 0.257, *C. carpio P* = 0.457, *R. rutilus P* = 0.511) between treatment and control groups across all species.

**Figure 3:**
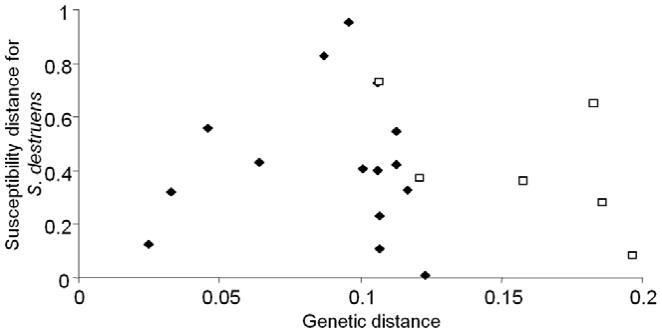
Host phylogeny and susceptibility to *Sphaerothecum destruens*. Genetic distance between all known susceptible species to *S. destruens* was plotted against the susceptibility distance to *Sphaerothecum destruens* for all the species combinations. The two families, *Cyprinidae* (□) and *Salmonidae* (♦) show different relationship patterns between genetic and susceptibility distances. Genetic distances were based in the pairwise analysis of ten Cytochrome b sequences. Analyses were conducted using the Tajima-Nei [30] method in MEGA4 [31]. All positions containing gaps and missing data were eliminated from the dataset. There were a total of 249 positions in the final dataset.

Overall, there was no significant correlation between the genetic distance and susceptibility matrices (Mantel statistic r = −0.0837, *P* = 0.67). Although not significant, a negative relationship between genetic distance and susceptibility appears to be present for the cyprinid family (Figure 3).

## Discussion

Our results characterise *S. destruens* as a generalist pathogen, with a range of potential host species as demonstrated by experimental exposures (Figures 1, 2; Table 1). In this study, *S. destruens* was detected in *A. brama*, *C. carpio* and *R. rutilus* following experimental infection with the parasite. *A. brama* experienced mortalities exceeding 50% when exposed to the parasite with 75% of these being positive for *S. destruens* in at least two of the three organs tested (Figure 1, Table 1). These results show that *A. brama* is highly susceptible to *S. destruens*. Mortality rate in the treatment group of *C. carpio* was considerably lower (8%), with lower infection prevalence (20%), suggesting that *C. carpio* is less susceptible to the parasite. However, following 28 d.p.e, there were only 50 *C. carpio* in the treatment and control groups. This could have potentially biased the estimated *S. destruens* prevalence.

In contrast, *R. rutilus* experienced high mortalities when exposed to *S. destruens* but with equivocal conclusions regarding its susceptibility as *S. destruens* was only detected at a prevalence of 5%. The observed discrepancy between mortality and disease prevalence could be due to parasite levels in the organs tested being lower than the nested PCR detection limit and/or differences in parasite tropism in this species; with *S. destruens* being more prevalent in organs other than those tested.

Although there were significantly higher mortalities in the groups exposed to *S. destruens* compared to control groups, fish died in the sham-exposed *A. brama* group. Most of these mortalities occurred during the first 15 d.p.e. (n = 11; 0/12 tested positive for *S. destruens*). This could be due to stress following the sham exposure. Mortalities were also observed during this period for the treatment group (treatment mortalities in first 15 d.p.e.: n = 12; 8/12 [67%] tested positive for *S. destruens*), however, in the case of the treatment group mortalities continued to increase past day 15.

*Sphaerothecum destruens* was not detected in the sampled *A. brama*, *C. carpio*, and *R. rutilus* at six and 11 months p.e. While prior exposure to *S. destruens* has never been reported from the source populations, surviving fish from the exposed group could have been naturally resistant to the parasite, being either refractory to initial infection or able to clear early stages of parasitism. Alternatively, surviving individuals might have entered a carrier state or developed a latent infection [22]. The absence of *S. destruens* in the surviving, experimentally-exposed fish likely suggests that either sterile immunity or resistance has occurred. There is no experimental evidence to suggest that the nested PCR used here is capable of detecting the carrier state [23]. Although not the focus of the current study, absence of carrier state should be confirmed via cohabitation of surviving fish with naïve individuals.

The low, but steady, mortality caused by this parasite (illustrated in Figure 2) highlights the danger in under detecting *S. destruens*'s related mortalities in the wild. Moribund fish are usually susceptible to predation and are thus not detected in the wild. In addition, occasional low mortality levels are often attributed to natural mortality and are thus not reported to the relevant agencies. Thus, although populations progressively decline and eventually collapse as shown in previous studies [6], the pathogen causing this decline remains undetected. It is also important to note that the factors leading to disease and mortality in a laboratory setting could differ from the ones acting in the wild. In the wild, the probability that individuals will be exposed to the minimum infectious dose could vary greatly and thus the impact on wild populations could be less severe compared to that in a laboratory setting. This highlights the need for longitudinal studies using wild populations which have or have not been exposed to *S. destruens* to assess whether this pathogen exerts a population level effect.

Among generalist parasites, some will preferentially exploit parasites from the same phylogenetic lineage whereas others appear to use a random set of locally available hosts [24]–[25]. The apparent lack of phylogenetic influence on host susceptibility to *S. destruens* suggests that this parasite belongs to the latter type of generalist parasites (Figure 3). It is possible that by exploiting a broader phylogenetic range of hosts, the parasite will use a number of locally available hosts and in doing so will maximise its survival and range expansion opportunities [24].

The effect of generalism on pathogenicity is unpredictable and is often not considered [23]. Generalist parasites can infect and cause high mortalities in hosts in which they do not have to persist indefinitely as long as they can persist in a reservoir host and even in the environment. Thus, in the absence of a strong host-parasite co-evolution generalist parasites can cause disease outbreaks. Such outbreaks can vary in frequency and magnitude with detrimental effects on the susceptible host (e.g. *Escherichia coli* O157 in humans) [25].

*Sphaerothecum destruens* is a true generalist with a highly invasive cyprinid (*P. parva*) as a reservoir host [6]. The rapid spread of *P. parva* through Europe via the aquaculture trade increases the risk of *S. destruens* introduction to a multitude of naïve fish communities enhancing the possibility for range expansion by this infectious parasite. The documented susceptibility and high mortalities in both salmonid and cyprinid species place *S. destruens* as a high risk parasite for freshwater biodiversity. In addition, these findings provide further illustration of the impacts of allochthonous infectious diseases on native fauna highlighting the risks associated with animal (and plant) trade at larger scales.

## Materials and Methods

### Ethics statement

All animal procedures followed strict guidelines set forward by the Home Office, UK. The project was approved by the Bournemouth University ethics committee and was performed under the Home Office licence no. 80/1979.

### Sources of parasite, spore purification and DNA extraction

*Sphaerothecum destruens* spores used in this challenge were originally isolated from wild *L. delineatus* [6] and were cultured *in vitro* in *Epithelioma papulosum cyprini* cells [26] as described in Andreou *et al.* [18].

The tissues collected from sampled experimental fish and mortalities included the kidney, liver, posterior intestine, gill and gonad (if present). DNA was extracted from each tissue separately (15 mg each) using the Qiagen DNeasy 96 Blood & Tissue kit (rodent tail protocol). All steps were performed according to the manufacturer's guidelines, with an overnight incubation at 55°C and elution volume of 150 µl. Extracted DNA was quantified in a spectrophotometer at 260 nm (NanoDrop ND-1000; Labtech) and stored at −70°C until further analysis.

*Sphaerothecum destruens* was detected using a nested PCR amplifying a segment of the 18S rRNA gene [23]. PCR products were migrated on a 1.5% agarose gel which was post-stained with ethidium bromide (0.5 µg/ml). An individual was scored positive if *S. destruens*-specific DNA was amplified from any of its organs tested.

### Fish source

*Abramis brama* and *R. rutilus* were supplied by the Calverton fish farm (Environment Agency, Calverton, Nottingham, UK). *Cyprinus carpio* originated from Water Lane fish farm (Burton Bradstock, Bridport). All fish were approximately one year old (1+) at the time of exposure to *S. destruens* spores. There has been no report of *S. destruens* infection in any of the farms. A total of 120 fish (60 exposed to *S. destruens* and 60 as controls) were used per species during the challenge experiments. The weight and length of ten randomly sampled fish per species were recorded at the onset of the experiment. Fish were kept in quarantine for 30 days prior to challenge with *S. destruens*.

Fish were fed twice a day with 1% of their body weight with CypriCo Crumble Astax (protein 53%, fat 13%, crude fibre 0.6%, ash 10.7%, astaxanthin 80 mg/kg; supplemented with vitamins A, D3, E and C; Coppens, Netherlands). All tanks had 25% of their water exchanged weekly and were checked for mortalities three times per day. Dead fish were collected and dissected immediately. Tissue samples were preserved for molecular analysis to test for the presence of *S. destruens*.

### Experimental Infection with *Sphaerothecum destruens*

Fish from *A. brama*, *C. carpio* and *R. rutilus* were divided into six replicate 70 L tanks each containing 20 fish per tank. Each tank had its own biological filter and was aerated using an air pump. Water temperature was kept constant at 20°C and the photoperiod was maintained at 16 h light and 8 h dark. The treatment group (n = 60) was divided into three holding tanks (n = 20 per tank). Similarly, the control group (n = 60) was divided into three holding tanks (n = 20 per tank)

The exposure protocol followed was adapted from the protocol used to expose *O. tshawytscha* to *S. destruens* [23]. Fish were exposed to *S. destruens* spores (average concentration 8.6×10^4^ spores ml^−1^) in eight litres of de-chlorinated 20°C water aerated using an air pump. Control fish were sham exposed. For each species, three separate exposures to *S. destruens* were performed at three-day intervals. Exposures were maintained for four hours. The end of the third exposure was considered as Time 0.

The sampling strategy for the treatment groups is detailed in Table 2. Sampling at six months and at the end of the experiment (11 months) was identical for the treatment and control groups. Fish were euthanized with an overdose of 2-phenoxylethanol and their weight and length was recorded. Fulton's condition index K*_F_* was calculated with the following formula [27]. Tissue samples were harvested and stored at −70°C for molecular analysis.

**Table 2:** Sampling strategy for the treatment groups *Abramis brama*, *Cyprinus carpio* and *Rutilus rutilus*.

Species	Mortalities			Sampled fish (6-months p.e.)			Surviving fish at 11 months p.e.		
	K, L, I	Gi,Go	N	K, L, I	Gi, Go	N	K, L, I	Gi, Go	n
*A. brama*	×		32	×	×	5	×		23
*C. carpio* *	×		5	×		5	×		40
*R. rutilus*	×	×**	22	×	×	5	×		33

List of organs and organ numbers which have been tested for the presence of *Sphaerothecum destruens* DNA. Organs tested included the kidney (K), liver (L), intestine (I), gill (Gi) and gonad (Go). n: number of fish sampled.

* : at 28 d.p.e. the liver, kidney and intestine of 10 *C. carpio* were tested for *S. destruens*.

** : gill and gonad tissues were analyzed in only 13 of the 22 *R. rutilus* mortalities.

### Statistical analysis

All statistical analyses were performed using SPSS 14.0 (SPSS Inc., Chicago, Illinois, USA) unless otherwise stated. Statistical significance was accepted when *P*≤0.05. Standard deviation of the mean was calculated. Disease prevalence was calculated as: (number of *S. destruens* positive fish/total number of fish tested)×100. Survival analysis (Kaplan–Meier survival curves and log rank tests) were calculated for the three cyprinid species investigated. The genetic and susceptibility distance matrices were correlated using the Mantel test available from the Vegan package [28] in R [29].

### Determining genetic and susceptibility distances

In order to investigate the phylogenetic influence of the host on the susceptibility to the parasite, genetic and susceptibility distances between susceptible host species were calculated. Genetic distance between susceptible species to *S. destruens* were calculated using the Tajima Nei genetic distance [30] using the software MEGA version 4 [31]. The Cytochrome b genetic marker was used to calculate genetic distances and sequences were obtained from NCBI GenBank (Table 3).

**Table 3:** GenBank sequences *Sphaerothecum destruens* prevalence values used in genetic and susceptibility distances.

Species	Cytochrome b	Prevalence	Exposure method	Ref.
Chinook salmon (*Oncorhynchus tshawytscha*)	AF392054	100, 71	Injection Water immersion	10, 15, 23
Coho salmon (*Oncorhynchus kisutch*)	AF165079	98	Injection	15
Rainbow trout (*Oncorhynchus mykiss*)	L29771	42.5	Injection	15
Atlantic salmon (*Salmo salar*)	AF133701	75	Disease outbreak (in aquaculture)	11
Brown trout (*Salmo trutta*)	X77526	43.3	Injection	15
Brook trout (*Salvelinus fontinalis*)	AF154850	2.6	Injection	15
Carp (*Cyprinus carpio*)	X61010	20	Water immersion	*^1^
Bream (*Abramis brama*)	Y10441	75	Water immersion	*
Roach (*Rutilus rutilus*)	Y10440	5	Water immersion	*
Sunbleak (*Leucaspius delineatus*)	Y10447	67, 40, 38	Cohabitation with *Pseudorasbora parva*	6, 17, 20

Mean prevalence was calculated and used where multiple prevalence values were available for a species. The infection method used is also provided.

(*) Current study.

Susceptibility distance was defined as the difference in susceptibility to *S. destruens* between known susceptible species to the parasite and was calculated by subtracting *S. destruens* prevalence values for all possible pairs of fish species. Mean prevalence values were used for species with more than one reported *S. destruens* prevalence value. Prevalence values for the Salmonidae were obtained from Hedrick *et al.* [11], Arkush *et al.* [15] and Mendonca and Arkush [23]. For example, in the case of the *O. tshawytscha* – *O. mykiss* pair, *O. tshawytscha* had a mean *S. destruens* prevalence of 85.5% and *O. mykiss* a 42.5% prevalence giving a susceptibility distance of 43% or 0.43 (Figure 3).
